# Effects of Daily Saskatoon Berry Supplementation on Cardiometabolic Health, Gut Microbiota, and Short-Chain Fatty Acids in Healthy Adults

**DOI:** 10.3390/ijms27083644

**Published:** 2026-04-19

**Authors:** Eunseo Lee, Amy Hui, Harvey Lee, Jiaan Sun, Garry X. Shen

**Affiliations:** 1 Department of Internal Medicine, University of Manitoba, Winnipeg, MB R3E 3P4, Canada; ellie.lee@umanitoba.ca (E.L.); amy.hui@umanitoba.ca (A.H.); jiaansun@hibrillant.com (J.S.); 2 Rossmere Medical Centre, Winnipeg, MB R2K 2M5, Canada; hblcorp@shaw.ca

**Keywords:** Saskatoon berry, healthy adults, cardiometabolic health, dietary fiber, gut microbiota, fecal short-chain fatty acids

## Abstract

Saskatoon berry (SB), a traditional food of Indigenous people, has been associated with cardiometabolic benefits in animal models; however, its effects on humans remain unclear. This study investigated the effects of dried SB consumption on cardiometabolic outcomes, gut microbiota, and short-chain fatty acids (SCFAs) profiles in healthy adults. In a 10-week, single-arm, and open-label trial, 20 healthy adults consumed 40 g/day of freeze-dried whole SB. Biochemical measures, physical exams, dietary records, participant feedback, and fecal samples were collected before and after the intervention. Gut microbiota composition and fecal SCFAs were profiled using 16S-rRNA sequencing and gas chromatography–mass spectrometry, respectively. SB intake significantly reduced fasting plasma glucose, total cholesterol (TC), low-density lipoprotein-cholesterol (LDL-c), non-high-density lipoprotein-cholesterol (non-HDL-c), systolic blood pressure, and high-sensitivity C-reactive protein, while increasing dietary fiber intake. Fiber intake was negatively correlated with TC, LDL-c and non-HDL-c (*p* < 0.05). The relative abundance of fecal Prevotellaceae increased after SB consumption and was positively correlated with multiple fecal SCFAs (*p* < 0.05–0.0001), while being negatively associated with lipid profiles and blood pressure. No adverse cardiovascular, hepatic, or renal dysfunction were observed; however, the significant increase in sugar intake may pose a risk for elevated blood glucose. Therefore, limiting other high-sugar foods during SB supplementation may be advisable for individuals with glucose intolerance. Overall, SB intake improved glucose and lipid metabolism and lowered blood pressure and inflammatory markers in healthy adults. These cardiometabolic benefits may be mediated by fiber and anthocyanins in SB and through modulation of gut microbiota and SCFA production; however, further confirmation is needed in subsequent randomized controlled trials.

## 1. Introduction

Saskatoon (*Amelanchier alnifolia*) is a deciduous shrub native to western Canada and the northwestern United States. Its fruit, commonly known as Saskatoon berry (SB), was a traditional food of Indigenous peoples in North America. SB was a classical component of pemmican, a traditional preserved food made with buffalo meat and berries [1,2]. Although SB is no longer routinely harvested in many Indigenous communities, it continues to be widely consumed across North America in the form of fresh fruit, juices, wine, jam or pie.

SBs are enriched with dietary fiber, essential micronutrients, vitamins, and polyphenolic compounds, particularly anthocyanins, which are known for their potent antioxidant and anti-inflammatory properties [3,4]. Preclinical studies demonstrated that dietary supplementation with dried SB powder or cyanidin-3-glucoside significantly reduced fasting plasma glucose (FPG), total cholesterol (TC), low-density lipoprotein-cholesterol (LDL-c), triglycerides (TG), and pro-inflammatory cytokine levels in mice with high-fat, high-sucrose diet-induced obesity. Additionally, SBs were previously shown to improve hepatic steatosis, restore gut microbiota composition, and enhance the production of short-chain fatty acids (SCFAs) in diet-induced insulin resistant mice [4,5,6,7]. Du Preez et al. demonstrated that SB supplementation lowered postprandial glucose, abdominal adiposity, blood pressure, and hepatic microvesicle size in high-fat diet-fed rats [8]. Moreover, Liu et al. reported the presence of SB-derived anthocyanins in plasma and urine samples from healthy volunteers, indicating the bioavailability of anthocyanins following SB ingestion in humans [9].

Together, these findings suggest that SB may be considered as a potential dietary supplement to reduce the risk of type 2 diabetes (T2D), metabolic syndrome, hypertension, and metabolic-dysfunction-associated steatotic liver disease (MASLD). Despite promising preclinical evidence in animal models, the metabolic benefits of SB have not been documented in human subjects.

To address this knowledge gap, we conducted a single-arm pilot study to evaluate the safety, acceptance, and feasibility of freeze-dried whole SB consumption in humans, as well as its potential cardiometabolic effects. Twenty healthy adults consumed SB for 10 weeks, and a broad range of clinical and biochemical outcomes was assessed before and after the dietary intervention, including fasting and postprandial plasma glucose levels, lipid profile, insulin resistance, blood pressure, inflammatory biomarker, gut microbiota and fecal SCFA compositions.

## 2. Results

### 2.1. Participants

**Figure 1:**
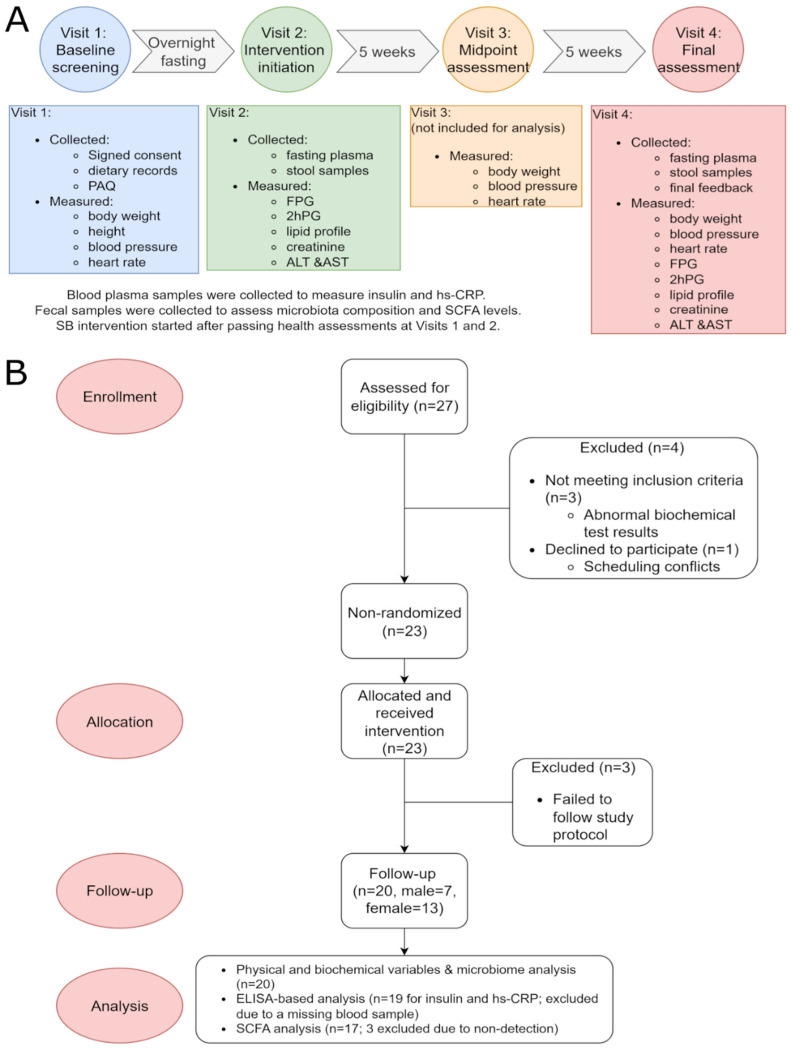
Study timeline and participant flowchart. (**A**) Overview of the office visits timeline. Abbreviations: PAQ: physical assessment questionnaire; FPG: fasting plasma glucose; 2hPG: 2 h post-load plasma glucose; ALT: alanine aminotransferase; AST: aspartate aminotransferase; hs-CRP: high-sensitivity C-reactive protein; SCFA: short-chain fatty acids. (**B**) Overview of participant enrollment and follow-up.

Of the 27 participants initially recruited, 3 were excluded due to abnormal baseline biochemical test results and 1 withdrew due to scheduling conflicts. Among the remaining 23 participants who began the intervention, 3 failed to adhere to the assigned dietary regimen and were excluded from the final analysis. A total of 20 participants (7 males and 13 females) completed the full regimen, corresponding to a dropout rate of 7/27 (26%). The average age of the participants was 38.45 ± 9.12 years, with a range of 22 to 59 years. A detailed study timeline and participant flowchart are presented in Figure 1.

### 2.2. Nutritional Profile of 40 g of SB

Table 1 summarizes the nutritional profile of a daily serving of freeze-dried whole SB (40 g). Each serving provided 133 kcal, 0.67 g of fat, 1.5 g of protein, 11.7 g of fiber, 20 g of sugar, and 1.7 mg of cholesterol, corresponding to 0.7%, 1.7%, 1.0%, 47.0%, 12.0%, and 0.6% of the recommended daily allowance (RDA) set by Health Canada [10], respectively. The total anthocyanin content in dried SB was 4.04 mg/kg containing cyanidin-3-galactoside (C3Ga; 70.2%) and cyanidin-3-glucoside (C3G; 17.2%). Each daily serving of SB provides 0.16 mg of total anthocyanin, including 0.11 mg of C3Ga and 0.028 mg of C3G.

### 2.3. Changes in Clinical, Lifestyle and Dietary Variables Following the Intervention

Clinical profiles and lifestyle patterns of participants at T0 and T10 are presented in Table 2, with dietary intakes shown in Table 3. After the intake of 40 g/day of whole dried SB for 10 weeks, significant reductions were observed in FPG, TC, LDL-c, non-HDL-c and systolic blood pressure (SBP) (*p*_(FDR)_ < 0.05; Table 2; Figure S1A–E). Notably, the levels of high-sensitivity C-reactive protein (hs-CRP), an inflammatory biomarker, were reduced by nearly 50% at T10 compared to T0 (*p*_(FDR)_ < 0.05; Table 2; Figure S1F).

Expression of values, % change and *p*_(DER)_ are the same as described in the footnote of Table 2.

Physical activity index, alcohol consumption, body mass index, diastolic blood pressure (DBP), heart rate, 2 h post-load plasma glucose (2hPG), creatinine, liver enzymes (AST and ALT), triglycerides, and HDL-c showed no significant change between T0 and T10 (Table 2). Insulin and insulin resistance (HOMA-IR) decreased by 9.58% and 13.30%, respectively, although these reductions did not reach statistical significance (Table 2).

Compared to baseline, the typical daily intakes of dietary fiber, sugar and vitamin C in the healthy subjects during SB intake were increased by 37.29%, 41.61% and 72.26%, respectively (*p*_(FDR)_ < 0.05; Figure S1G–I).

### 2.4. Correlations Among Metabolism-Related Clinical, Biochemical and Nutritional Variables

**Figure 2:**
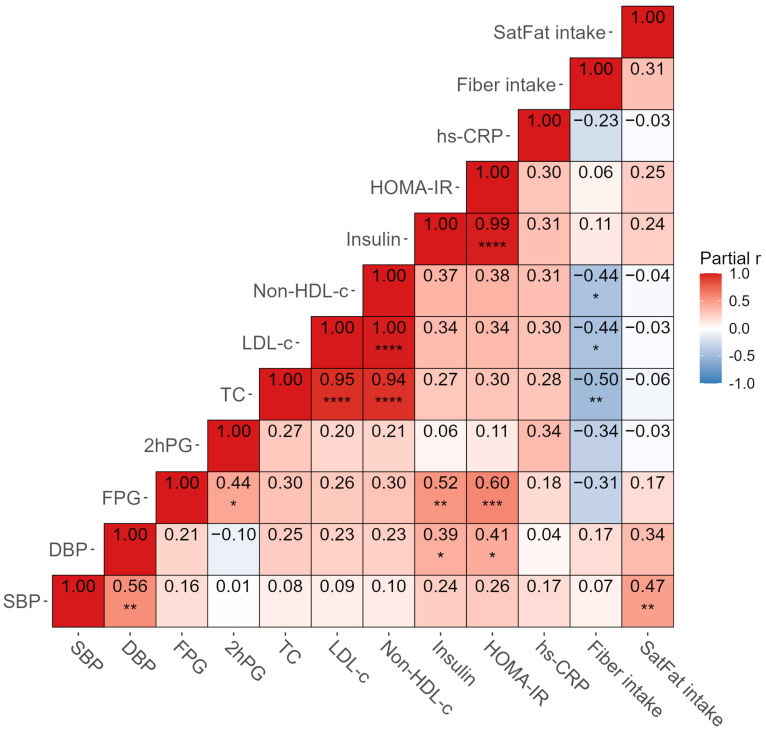
Associations among clinical and dietary variables. Abbreviations: SBP: systolic blood pressure; DBP: diastolic blood pressure; FPG: fasting plasma glucose; 2hPG: 2 h post-load plasma glucose; TC: total cholesterol; LDL-C: low-density lipoprotein cholesterol; non-HDL-c: non-high-density lipoprotein-cholesterol; HOMA-IR: homeostasis model assessment of insulin resistance; hs-CRP: high-sensitivity C-reactive protein; SatFat: saturated fat. Cell colors and numbers indicate Pearson’s partial r adjusted for age and sex covariates. Correlations involving insulin, HOMA-IR, and hs-CRP were performed using 39 samples, while all other correlations were performed using 40 samples. *: *p*_(FDR)_ < 0.05; **: *p*_(FDR)_ < 0.01; ***: *p*_(FDR)_ < 0.001; ****: *p*_(FDR)_ < 0.0001.

Figure 2 illustrates correlations among clinical, biochemical and dietary variables. Strong positive associations were observed among TC, LDL-c, and non-HDL-c (*p*_(FDR)_ < 0.0001), as well as among FPG, insulin, and HOMA-IR (*p*_(FDR)_ < 0.01–0.0001). FPG was also positively correlated with 2hPG (*p*_(FDR)_ < 0.05). Lipid profiles (TC, LDL-c, and non-HDL-c), FPG, and 2hPG negatively correlated with fiber intake, yet only the correlations between fiber intake and lipid profiles showed significance (*p*_(FDR)_ < 0.05–0.01). DBP and saturated fat intake were positively correlated with SBP (*p*_(FDR)_ < 0.01).

### 2.5. Effect of SB Intake on Fecal SCFAs and Correlations Among SCFA Concentrations, Clinical, and Dietary Variables

**Figure 3:**
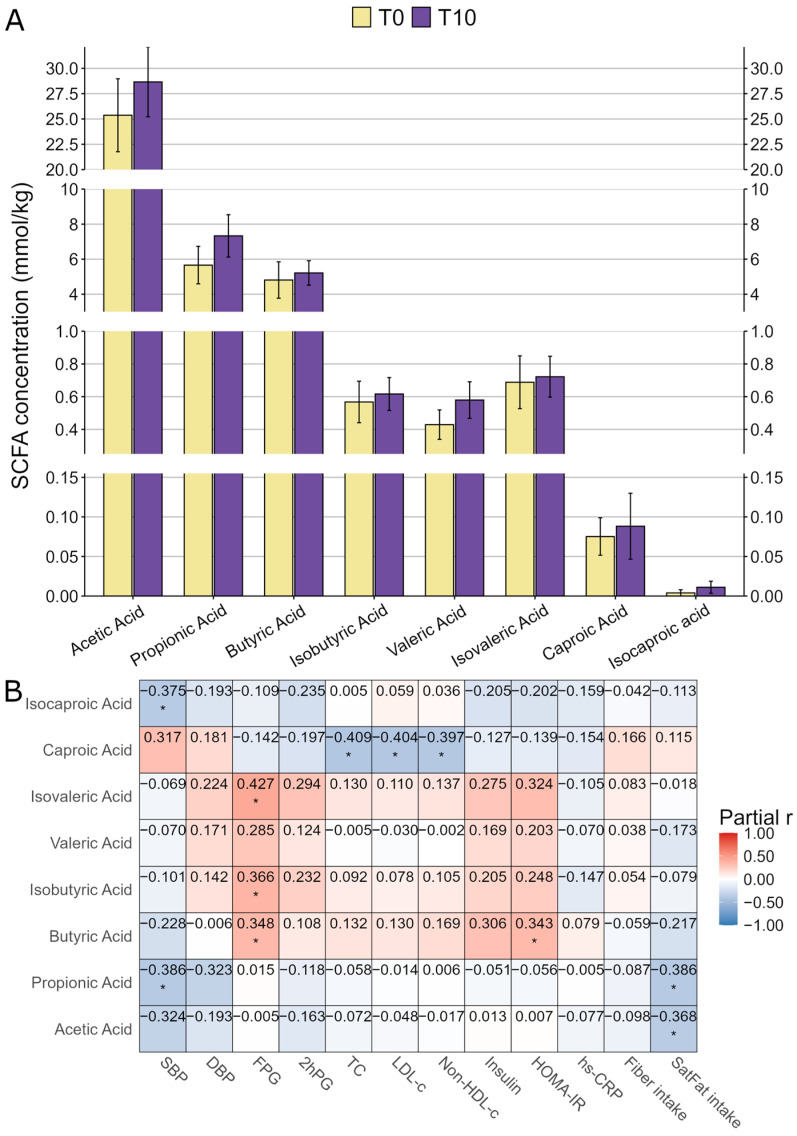
Fecal SCFA concentrations and their associations with SB intake, clinical, and dietary variables. Abbreviations: SCFA: short-chain fatty acids. (**A**) Fecal SCFA concentrations at baseline (T0) and after 10 weeks of SB treatment (T10). Comparisons were performed using 17 paired samples (n = 17 pairs; total observations = 34), rather than 20 pairs, because SCFA data were missing for three samples. Values were presented in mean ± SE. (**B**) Correlation matrix showing associations between clinical and dietary variables and SCFA levels in the healthy participants. All available samples with SCFA data were included (n = 37). Cell colors and numbers indicate Pearson’s partial r adjusted for age and sex covariates. *p*-values were unadjusted due to the exploratory nature of the analysis. *: *p* < 0.05. Abbreviations: SBP: systolic blood pressure; DBP: diastolic blood pressure; FPG: fasting plasma glucose; 2hPG: 2 h plasma glucose after 75 g oral glucose intake; TC: total cholesterol; LDL-c: low-density lipoprotein-cholesterol; non-HDL-c: non-high-density lipoprotein-cholesterol; HOMA-IR: homeostasis model assessment of insulin resistance; hs-CRP: high-sensitivity C-reactive protein; SatFat: saturated fat.

Concentrations of all eight different types of fecal SCFAs were moderately, but not significantly, elevated after the SB intervention (Figure 3A). Correlations between SCFAs and clinical and dietary variables are shown in Figure 3B. SBP was negatively correlated with propionic and isocaproic acids (*p* < 0.05), while FPG was positively correlated with butyric, isobutyric, and isovaleric acids (*p* < 0.05). TC, LDL-c, and non-HDL-c were negatively correlated with caproic acid (*p* < 0.05). HOMA-IR was positively correlated with butyric acid (*p* < 0.05), whereas saturated fat intake was negatively correlated with acetic and propionic acids (*p* < 0.05).

### 2.6. Impact of SB Intervention on Gut Microbiota and Its Associations with SCFAs, Clinical Outcomes, and Dietary Intake

**Figure 4:**
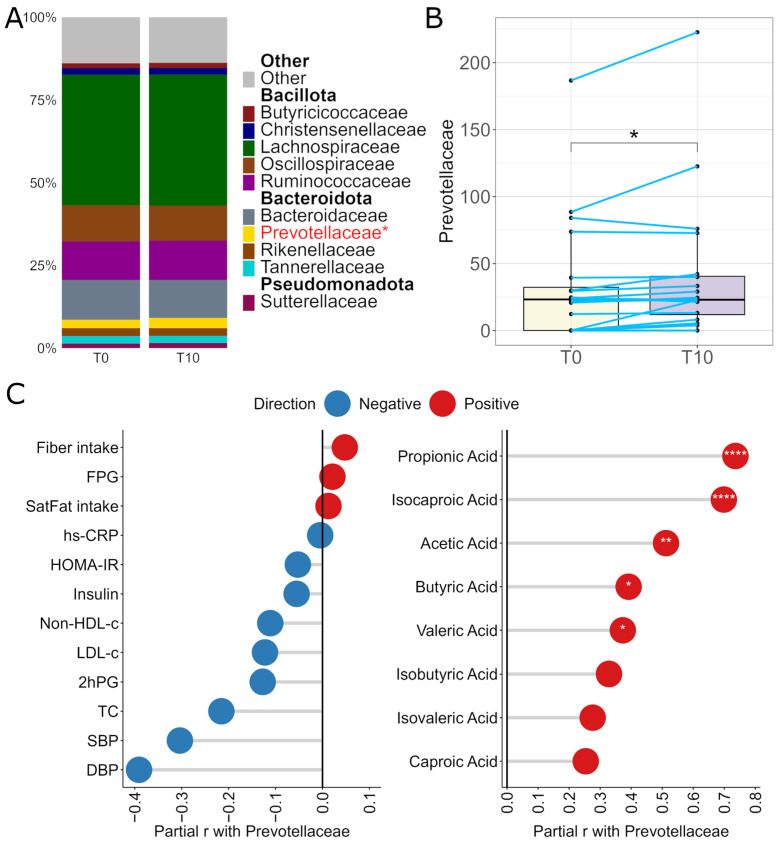
Associations of the gut bacterial family Prevotellaceae with SB intake, clinical and dietary variables, and SCFAs. Abbreviations: T0: baseline; T10: 10 weeks post-intervention. (**A**) Relative abundance of gut microbiota at the family level in pre- (T0) and post-dietary (T10) intervention groups. Data are presented for the 10 most abundant family bacteria. (**B**) Paired comparisons of Prevotellaceae relative abundance between T0 and T10 in boxplots with blue lines connecting data from the same individuals. Statistical significance was calculated via Wilcoxon signed-rank test (*: *p* < 0.05 & *p*_(FDR)_ < 0.20, n = 20/group). (**C**) Pearson’s partial r between Prevotellaceae relative abundance, clinical and dietary variables, and SCFA concentrations. Abbreviations: SBP: systolic blood pressure; DBP: diastolic blood pressure; TC: total cholesterol; FPG: fasting plasma glucose; 2hPG: 2 h plasma glucose after 75 g oral glucose intake; HDL-c: high-density lipoprotein cholesterol; LDL-c: low-density lipoprotein-cholesterol; Non-HDL-c: non-high-density lipoprotein-cholesterol; HOMA-IR: homeostasis model assessment of insulin resistance; hs-CRP: high-sensitivity C-reactive protein; SatFat: saturated fat. Cell colors indicate Pearson’s partial r adjusted for age and sex covariates. *: *p*_(FDR)_ < 0.05; **: *p*_(FDR)_ < 0.01; ****: *p*_(FDR)_ < 0.0001 (sample sizes were n = 37 for analyses involving SCFAs, n = 39 for HOMA-IR, insulin, and hs-CRP and n = 40 for all other variables).

The α- and β-diversities of the gut microbiota did not significantly differ between the T0 and T10 groups (Figure S2A,B). Relative abundances were examined for the 20 most abundant genera and 10 most abundant families (Figure S2C and Figure 4A). Only the relative abundance of Prevotellaceae family significantly increased after 10 weeks of SB intervention compared to that of T0 (*p* < 0.05 & *p*_(FDR)_ < 0.20; Figure 4B). The relative abundance of Prevotellaceae significantly correlated with valeric, butyric, acetic, isocaproic, and propionic acids (*p*_(FDR)_ < 0.05–0.0001) and negatively associated with DBP, SBP, TC, LDL-c, non-HDL-c, 2hPG, insulin, HOMA-IR, and hs-CRP (Figure 4C).

### 2.7. Participants’ Feedback on Dietary Intervention

Table 4 presents the follow-up survey results at the end of the study. Sixteen out of twenty participants (80%) submitted the survey and 81% of them reported no difficulty consuming 40 g of dried SB daily. Half of them consumed the dried SB in the afternoon, and 37% in the morning. Most participants (81%) administered dried SB as a snack and 13% consumed it with cereal. Over half of participants (56%) ate the assigned berry every day, 38% missed 1–2 days, and 6% missed 3–4 days during the trial. Most participants liked the texture (87%) and taste (94%) of dried SB. More than half of the participants felt physical or behavior changes, including weight loss, bowel habit and less sugar craving. The most common change was an increase in bowl movement frequency (62%). In addition, 94% of participants indicated that they would participate in or recommend their relatives or friends to join a follow-up study on SB.

## 3. Discussion

The health-promoting effects of various edible berries, including strawberry, blueberry, blackberry, cranberry, and raspberry, have been extensively investigated in both animal models and human subjects [12,13,14]. In contrast, fewer studies have evaluated the health benefits of SB [3,4,5,6,7,8]. Furthermore, the only human study addressed the bioavailability of its components, without examining SB’s effects on metabolism [9]. In the present study, we report novel findings on the metabolic benefits of SB intake in healthy adults. Daily consumption of 40 g of dried SBs for 10 weeks was associated with cardiometabolic and anti-inflammatory benefits in healthy adults, without any evident adverse effects. These effects were accompanied by alterations in gut microbiota composition and fecal SCFA levels.

Both FPG and 2hPG are commonly used for screening abnormalities and diagnosing diabetes [15]. In the present study, daily SB intake reduced FPG (−4.2%; *p* < 0.05) and 2hPG (−3.5%), while significantly increasing fiber intake (+32.7%; *p* < 0.05) in healthy subjects. These findings suggest that SB may lower blood glucose levels, potentially through a substantial increase in dietary fiber intake [16]. The results are consistent with a prior study by Xiao et al., which demonstrated that fiber-rich red raspberry improved glucose modulation in individuals with overweight or prediabetes [17]. Furthermore, the administration of dried SB introduced considerable amounts of anthocyanins, a class of flavonoids that have been shown to improve glucose metabolism in insulin-resistance hepatocytes [18] and in diet-induced diabetic mouse models [4,5,6,7,18]. Previous clinical study has demonstrated that a fiber-rich diet reduces circulating glucose levels in patients with type 2 diabetes [19]. Consistent with this finding, increased fiber intake may have partially linked to the glucose-lowering effect of SB in healthy adults. In addition, the results of the present study demonstrated that dried SB intake lowered FPG in healthy adults, even with increased sugar intake and without a rise in plasma insulin. Bioactive compounds enriched in SB, including anthocyanins and fiber, may act synergistically to improve glucose homeostasis in healthy adults. This hypothesis remains to be verified in future studies.

The results of the present study demonstrated that SB intake led to significant reductions in TC, LDL-c, and non-HDL-c, suggesting its potential to lower cholesterol synthesis in healthy subjects. These findings are consistent with previous animal studies, where 10–15 weeks of SB powder intake reduced TC and LDL-c in the high-fat, high-sucrose diet-fed mice [4,5,6,7]. A potential contributor to the cholesterol-lowering effect of SB is the dietary fiber provided by SB consumption. The daily dosage of SB in the present trial provides 11.7 g of fiber/day, which is equivalent to 18–47% of the recommended daily fiber allowance among people in various age groups [10]. Fiber intake negatively correlated with TC, LDL-c and non-HDL-c in the present study, which further suggests that fiber may play an important role in decreasing apolipoprotein (apo) B-rich lipoproteins-cholesterol in healthy adults. These findings support the results from a recent clinical study reporting an inverse association between dietary fiber intake and circulating cholesterol in 100 volunteers on a low-carbohydrate, high-fat diet [20]. The molecular target of SB on cholesterol synthesis or the assembly of non-HDL-cholesterol lipoproteins remains to be identified.

In line with a previous study reporting that SB intake lowered SBP and DBP in high-fat diet-fed rats [8], the present study found a significant reduction in SBP following 10 weeks of SB administration. This effect may partially be explained by the significant positive correlation between saturated fat intake and SBP. Post-intervention feedback revealed that most participants consumed SB as a snack, potentially replacing the saturated fat-rich snack options such as potato chips, which may have reduced saturated fat intake and lowered blood pressure. Indeed, a notable decrease in saturated fat consumption (−16.6%) was detected at T10, although this change did not reach statistical significance. The link between saturated fat and SBP is consistent with the findings from epidemiological studies in middle-aged and older American women, as well as Mediterranean diet trials showing that dietary fat influences blood pressure [21,22]. This is further supported by a systematic review indicating that eating less saturated fat may attenuate risk of cardiovascular diseases [23]. Thus, the decline in saturated fat intake and blood pressure could represent a cardiovascular benefit of SB.

Hs-CRP is a well-recognized biomarker of chronic inflammation and is commonly used to assess metabolic and cardiovascular disease risk in clinical trials [24,25]. In our study, hs-CRP levels in healthy adults were decreased by 49.1% following SB intervention, reinforcing a strong anti-inflammatory potential of SB in humans, as previously demonstrated by diet-induced diabetic rodent models [4,5]. In those animal studies, anthocyanins were identified as key compounds that inhibit vascular inflammation and reduce endothelial adhesion molecule expression [26]. Notably, SB contains higher anthocyanin levels than other common berries [27], with the daily dose of 40 g SB providing approximately 0.16 mg of total anthocyanins. Alongside animal evidence [3,4,5,7], our findings in healthy subjects align with trials on red-fleshed apples and other polyphenol-rich fruits, which reported reductions in hs-CRP and LDL-c in hypercholesterolemic patients [28,29]. These findings support a role for SB in mitigating chronic low-grade inflammation in healthy adults, which may reduce the risk of cardiometabolic diseases.

Herein, we report that SB intake significantly increased Prevotellaceae, a gut microbial family positively associated with multiple SCFAs, as well as dietary fiber intake. Previous clinical studies revealed that eating mixed berries and strawberries can modulate gut microbiota composition and metabolites in healthy subjects [30,31]. The in vitro fermentation of Changyanning, a traditional herbal compound formula used to treat inflammatory digestive diseases, selectively increased the abundance of certain gut genera and SCFAs, including *Prevotella-9* and propionic acid, in a simulated digestion model [32]. In line with those findings, our results suggest that SB intake may similarly influence gut microbial composition and microbial metabolites in healthy adults. Prevotellaceae was also negatively associated with blood pressure, lipid profiles and 2hPG levels. These findings suggest that SB intake may influence cardiometabolic outcomes via increasing relative abundance of Prevotellaceae in the gut. Propionic and isocaproic acids, which were moderately elevated following the dietary intervention, exhibited negative correlations with SBP (*p* < 0.05) and were positively associated with Prevotellaceae (*p*_(FDR)_ < 0.0001). As SCFAs are products of microbial fermentation, this association suggests a pathway whereby SB increased the abundance of Prevotellaceae and SCFA production in the gut, which may subsequently contribute to reduced blood pressure. Together, these findings are consistent with prior reports linking SCFAs to blood pressure regulation [33] and support a potential role of SCFAs as mediators of blood pressure reduction in healthy adults regularly consuming SB.

SB consumption significantly increased sugar intake, as a daily serving of 40 g of SB accounts for close to 12 g of sugar/day, representing 12% of the RDA. This may suggest a potential risk of elevated blood glucose levels, particularly in individuals with impaired glucose regulation. Although SB intake did not increase FPG or 2hPG in healthy adults, limiting the intake of other high-sugar foods during SB administration should be recommended for individuals with glucose intolerance. No adverse effect on liver, kidney or cardiovascular function was observed during the intervention, supporting the safety of SB in healthy adults. Post-trial feedback indicated that the dried SB regimen was well tolerated and accepted by the healthy adult participants.

Several limitations of the results of the present study warrant discussion. This was a non-randomized, single-arm pilot study with moderate sample size, which may limit the power to distinguish SB-specific effects from temporal, seasonal, or behavioral confounders. In addition, the intervention used a single SB dosage and time point, without examining dose–response relationship or effects of intervention duration. Given the small sample size, adjustment in the correlation analyses was restricted to age and sex to reduce the risk of overfitting; thus, other potential confounders such as dietary intake and physical activity may have influenced the observed associations. The participants in this study were healthy adults, and the dosage and safety of dried SB in children, elderly, or pregnant individuals remain to be determined. Additionally, the gender distribution in this pilot study was unequal and was skewed towards women. Despite these constraints, this study provides novel findings on SB to guide the design of more rigorous trials in populations beyond healthy adults. Larger randomized controlled trials are needed to verify the effects of SB detected in the present study, determine optimal dosages and duration of intervention, long-term safety, and compliance in healthy subjects across different age groups in healthy subjects, as well as in metabolically compromised populations (e.g., prediabetes, T2D, hypertension and MASLD).

### 3.1. Conclusions

This study provides the first clinical evidence that the consumption of 40 g/day of dried SB for 10 weeks effectively lowered FPG and apoB-rich lipoprotein-cholesterol, inflammatory biomarkers and blood pressure in healthy adults, which was associated with an improvement in gut microbiota composition and SCFA production. No adverse effects were observed, and the regimen was well accepted by the participants; however, the increased sugar intake from SB may affect blood glucose levels. Although SB supplementation increased sugar intake, it did not raise fasting or 2 h postprandial plasma glucose levels in healthy adults. Nevertheless, limiting other sugar-rich foods and beverages during SB supplementation may be advisable for individuals with glucose intolerance. Overall, this pilot study indicates that dried SB could potentially reduce cardiometabolic risk in healthy adults as a dietary supplement.

### 3.2. Implications and Future Directions

The results suggest that dried SB is a well-tolerated, functionally active dietary supplement with the potential to reduce multi-cardiometabolic risks mediated by increased dietary fiber, anthocyanins, and the modulation of gut microbiota composition and SCFA production. These findings provide a strong rationale for conducting large-scale randomized controlled trials to explore the effects of dried SB in other healthy and clinically relevant populations and to evaluate its integration into broader dietary strategies for chronic disease prevention.

## 4. Methods

### 4.1. Study Design and Subjects

For this 10-week, single-arm, and open-label trial, 27 healthy volunteers (8 males and 19 females) were recruited from the students and staff of the University of Manitoba. Eligibility criteria included: (i) healthy adults; (ii) without diabetes, obesity, cardiovascular or hepatic disease; and (iii) voluntarily sign an informed consent. Exclusion criteria included: (i) currently pregnant or nursing and (ii) use of antibiotic, anti-diabetic, antihypertensive, anti-obesity, lipid-lowering medications or probiotic supplement within the last 3 months. Participants were considered “healthy” based on an initial self-report indicating no history of metabolic, cardiovascular, hepatic, or renal disease. Eligibility was subsequently confirmed through physician-reviewed clinical and biochemical screening, including BMI, blood pressure, heart rate, fasting plasma glucose, 2 h postprandial glucose, serum creatinine, AST, ALT, total cholesterol, triglycerides, HDL-c, LDL-c, and non-HDL-c. Participants with abnormal values were excluded. Of the 27 participants initially recruited, 3 were excluded due to abnormal baseline biochemical test results and 1 withdrew due to scheduling conflicts. Of the remaining 23 participants who began the intervention, 3 failed to adhere to the assigned dietary regimen and were excluded from the final analysis. In total, 20 participants (7 males, 13 females) completed the full regimen and were included in the final analysis (n = 20), corresponding to a dropout rate of 7/27 (26%). Power assessment was not conducted prior to the study because this was the first pilot trial evaluating the effects of SB in humans. Instead, the sample size of the trial was decided based on the recommendation from an overview of previous studies, which suggests that, for a continuous outcome, a sample size of 12–35 per group is recommended for pilot studies [34].

### 4.2. Ethics Approval and Trial Registration

The research protocol and informed consent were approved by the Biomedical Research Ethics Board in the University of Manitoba (HS23500) and the trial was registered at ClinicalTrials.gov (http://ClinicalTrials.gov, NCT04809688, accessed on 18 March 2026).

### 4.3. Dietary Supplement

Freeze-dried whole SBs (Martin cultivar) were obtained from Prairie Berries Inc. (Keeler, SK, Canada). SBs were harvested from local orchards, cleaned, and freeze-dried at −70 °C without any additive or preservative. Safety evaluation, including toxicity and contaminant testing, was completed by the manufacturer through customs inspection service. Dried SBs were stored in vacuum-sealed bags at −4 °C. Anthocyanins and anthocyanidins components in SB were quantified using high-performance liquid chromatography–mass spectrometry as previously described [35]. Other nutritional information, such as calories, fat, protein, and dietary fiber, were obtained from the nutrition label provided on the commercially packaged freeze-dried SB product from Prairie Berries Inc.

### 4.4. Dietary Regimen

Participants consumed 40 g of dried SBs/day for 10 consecutive weeks. Participants were instructed to maintain their usual diet and activity habits during the intervention.

### 4.5. Office Visits and Data Collection

Visit 1: Participants signed informed consent and completed a physical activity questionnaire (PAQ). Baseline physical assessments, including body weight, height, blood pressure, and heart rate, were conducted. Instructions were given for overnight fasting, 3-day dietary intake recording, and stool sample collection using stool nucleic acid collection and preservation tubes (Cat. 45660, Norgen Biotek, Thorold, ON, Canada) for visit 2.

Visit 2: After an overnight fast, blood samples were collected in the morning to assess plasma glucose, lipid profile, creatinine, alanine aminotransferase (ALT), and aspartate aminotransferase (AST). Participants then underwent a 75 g oral glucose tolerance test (OGTT). Aliquots of fasting plasma samples from each visit were stored at −70 °C for subsequent analysis of insulin and hs-CRP. Stool samples collected one day prior to the commencement of SB intervention were submitted during the visit and stored at −70 °C. Each participant received a 10-week supply of SB and was instructed to begin the consumption on the day after the confirmation of normal biochemical results.

Visit 3: At week 5 of the intervention, participants returned for an evaluation of adherence and general well-being on the dietary intervention. Physical assessments were repeated as those in Visit 1. Participants received stool sample collection kits to be collected at visit 4.

Visit 4: At the end of week 10, OGTT and blood withdrawal for other tests were conducted as in Visit 2. Post-intervention stool samples were collected and stored. PAQ and dietary intake records were obtained, and post-study feedback was collected.

### 4.6. Biochemical Analysis

Venous blood collection, OGTT, and standard clinical chemistry assays for TC, TG, LDL-c, HDL-c, non-HDL-c, ALT, AST, and creatinine were conducted by staff in the Department of Clinical Chemistry at the Health Sciences Centre, Winnipeg. Plasma concentrations of insulin and hs-CRP were quantified using commercially available ELISA kits from Crystal Chem (Insulin: Cat. #90095; hs-CRP: Cat. #80955, Itasca, IL, U.S.A.). Absorbance readings from ELISA tests were measured using an Agilent BioTek Epoch microplate spectrophotometer (Agilent Technologies, Winooski, VT, U.S.A.). Homeostatic model assessment for insulin resistance (HOMA-IR) was calculated using the formula as previously described: HOMA-IR = [Fasting insulin (µU/mL) × Fasting glucose (mmol/L)]/22.5 [36]. One plasma sample was lost during the collection-to-storage process. Consequently, this sample was excluded for correlation analyses of ELISA-based measures (insulin, HOMA-IR, and hs-CRP).

### 4.7. Food Intake, Physical Activity Assessment, and Feedback Collection

Self-reported daily dietary intake at baseline (T0) and study end (T10) was analyzed using the Elizabeth Stewart Hands and Associates (ESHA) Food Processor software (ESHA Research, Beaverton, OR, U.S.A.; version 11.14). Physical activities recorded at T0 and T10 collected from PAQ were quantified using a validated Physical Activity Index based on frequency, time and intensity of activities as previously described [11]. Feedback on taste, acceptability, and perceived effects on the SB supplementation was collected from participants at the end of the study via a standardized survey.

### 4.8. DNA Extraction and 16S rRNA Sequencing for Fecal Samples

DNA was extracted from the stool samples using the QIAamp PowerFecal DNA Kit (QIAGEN, Hildon, Germany) and sequenced at the Integrated Microbiome Resource at Dalhousie University. The V4–V5 region of the 16S rRNA gene was amplified using primers 515F (5′-GTGYCAGCMGCCGCGGTAA-3′) and 926R (5′-CCGYCAATTYMTTTRAGTTT-3′). Amplicons were normalized using the Just-a-Plate 96-well normalization kit (Charm Biotech, San Diego, CA, U.S.A.) and sequenced on an Illumina MiSeq platform (Illumina, San Diego, CA, U.S.A.) [37].

### 4.9. Amplicon Sequence Variants (ASV) Generation

Demultiplexed paired-end sequences were processed using QIIME2 (version 2024.10) [38]. Primer sequences were removed with Cutadapt [39], and sequence denoising was performed using DADA2 [40], both implemented in QIIME2, to generate ASVs. Taxonomy was assigned using a region-specific classifier trained with RESCRIPt [41] in QIIME2 on the SILVA 138.2 SSU-nr99 reference database [42]. Sequences identified as chloroplasts or mitochondria were removed prior to downstream analysis.

### 4.10. Quantification of SCFAs

Fecal SCFAs were extracted using propyl chloroformate and derivatized with a reaction system containing water, propanol and pyridine. Consequent analyses were performed using an Agilent 7890A (Santa Clara, CA, U.S.A.) gas chromatography linked with an Agilent 5975A inert XL EI/CI mass spectrometry in Microbiome Insights Inc. (Vancouver, BC, Canada) through customer service, as previously described [43]. In brief, SCFAs in stool samples were measured as absolute concentrations (mM) using external calibration curves created from genuine SCFA standards. To account for analytical variability, concentrations were normalized relative to the internal standard (2-ethylbutyric acid). Stool samples were mechanically homogenized before extraction to make sure the matrix was completely broken up. Quantification of SCFAs was performed using Chromeleon chromatography software (Thermo Fisher Scientific, version 7.2.10.23925), and the limit of detection for individual SCFAs under the described gas chromatography Flame Ionization Detector conditions was approximately 0.15625 mM. The limit of quantification was defined as the lowest calibration point meeting acceptable linearity and signal-to-noise criteria.

### 4.11. Gut Microbiota Profiling and Statistical Analysis

All data analyses were conducted in R platform (version 4.3.3). *p*-values were adjusted using the Benjamini–Hochberg method and are indicated as *p*_(FDR)_. Pairwise pre- and post-dietary intervention comparisons were performed using *t*-tests, with the Wilcoxon signed-rank test applied to variables with skewed distributions.

ASV table was imported as phyloseq objects [44]. To reduce noise, features with <0.1% relative abundance in fewer than two samples were removed, resulting in 596 taxa across 40 samples. Sequencing data were then normalized using total sum scaling followed by log transformation, except for α-diversity analyses, where rarefaction was applied using the lowest sample read count of 26,568. α-diversity was calculated using the Shannon index and phylogenetic diversity. β-diversity was examined using Bray–Curtis dissimilarities and distance-based redundancy analysis (db-RDA), adjusted for age and sex covariates with 9999 permutations for significance testing. Sequences were agglomerated at the genus and family taxonomic rank.

The 20 most abundant genera and the 10 most abundant families were included in the microbiota analyses. Among these, differential abundance analysis was performed using Wilcoxon signed-rank test, and taxa with raw *p* < 0.05 and *p*_(FDR)_ < 0.20 were considered significantly different between the pre- and post-intervention groups.

All correlation analyses were performed using Pearson partial correlation adjusted for age and sex, with *p*-values corrected for multiple testing. The only exception was the correlation between fecal SCFA concentrations and clinical or dietary variables, where unadjusted *p*-values were reported due to the exploratory nature of the analysis.

SCFA data were missing for three samples due to insufficient sample volume remaining after other analyses; therefore, comparative analyses of SCFA concentrations were performed on 17 paired samples (n = 17 pairs; total observations = 34), rather than 20 pairs. For SCFA correlation analysis, all available samples with SCFA measurements were included (n = 37). Concentrations of SCFAs in pre- and post-dietary intervention groups were compared using the Wilcoxon signed-rank test.

## Figures and Tables

**Table 1:** Nutrients in 40 g of freeze-dried whole Saskatoon berry.

Nutrients	Contents in 40 g Dried SB	% of Recommended Daily Allowance
Calorie	133	0.7
Fat (g)	0.67	1.7
Protein (g)	1.5	1.0
Carbohydrate	31.7	12.7
Fiber (g)	11.7	47.0
Sugar (g)	20.0	12.0
Cholesterol (mg)	1.7	0.6
Sodium (mg)	25	3.0
Potassium (mg)	375	11.0
Calcium (mg)	83.3	8.3
Iron (mg)	2.08	11.7
Magnesium (mg)	50.0	11.7
Manganese (mg)	0.83	36.7
Vitamin C (mg)	4.2	4.6
Vitamin E (mg)	0.33	1.7
Total anthocyanins (CGE mg)	0.16	NA
Cyanidin-3-galactoside (mg)	0.11	NA
Cyanidin-3-glucoside (mg)	0.028	NA

Abbreviations: CGE: cyanidin-3-glucoside equivalents. NA: not available in Health Canada database. The concentrations of anthocyanins were measured in the Department of Food and Human Nutritional Sciences at the University of Manitoba. The recommended daily allowance of macronutrients was obtained from Health Canada [10].

**Table 2:** Clinical and lifestyle variables of healthy participants before and after the dietary intervention.

Variables	T0	T10	Change (%)	*p*_(FDR)_
BMI (kg/M^2^)	23.20 ± 3.40	22.91 ± 3.43	−1.23	0.223
SBP (mmHg)	117.8 ± 11.35	111.5 ± 12.12	−5.35	0.029 *
DBP (mm/Hg)	71.00 ± 6.73	68.85 ± 6.84	−3.03	0.274
Heart rate (beats/min)	70.75 ± 9.39	74.00 ± 10.85	4.59	0.089
FPG (mM/L)	5.12 ± 0.47	4.90 ± 0.42	−4.24	0.020 *
2hPG (mM/L)	5.10 ± 1.47	4.92 ± 1.19	−3.53	0.423
Creatinine (μM/L)	72.80 ± 18.06	73.35 ± 17.90	0.76	0.677
AST (unit/L)	23.55 ± 13.10	20.90 ± 8.25	−11.25	0.153
ALT (unit/L)	22.60 ± 14.36	19.00 ± 11.25	−15.93	0.162
Total cholesterol (mM/L)	4.77 ± 0.92	4.56 ± 0.86	−4.31	0.017 *
Triglycerides (mM/L)	0.78 ± 0.23	0.69 ± 0.15	−12.11	0.063
HDL-c (mM/L)	1.70 ± 0.35	1.65 ± 0.35	−3.11	0.299
LDL-c (mM/L)	2.72 ± 0.92	2.60 ± 0.85	−4.70	0.031 *
Non-HDL-c (mM/L)	3.07 ± 0.98	2.92 ± 0.87	−4.65	0.034 *
Insulin (mU/L)	5.52 ± 4.65	4.99 ± 4.37	−9.58	0.269
HOMA-IR	1.29 ± 1.17	1.12 ± 1.06	−13.30	0.144
hs-CRP (μg/mL)	3.81 ± 4.05	1.94 ± 2.22	−49.07	0.024 *
Physical Activity Index	1.70 ± 0.47	1.75 ± 0.44	2.94	0.577
Alcohol (service/day)	0.50 ± 1.32	0.45 ± 1.23	−10.00	0.330

Abbreviations: T0: baseline; T10: 10 weeks post-intervention; BMI: body mass index; SBP: systolic blood pressure; DBP: diastolic blood pressure; FPG: fasting plasma glucose; 2hPG: 2 h plasma glucose after 75 g oral glucose tolerance test (OGTT); AST: aspartate transaminase; ALT: alanine transaminase; HDL-c: high-density lipoprotein-cholesterol; LDL-c: low-density lipoprotein-cholesterol; Non-HDL-c: non-HDL-cholesterol; HOMA-IR: homeostasis model assessment of insulin resistance; hs-CRP: high-sensitivity C-reactive protein. Sample sizes: n = 19/group for insulin, HOMA-IR, and hs-CRP; n = 20/group for all other variables. Values are expressed as mean ± standard deviation at T0 and T10. Change represents percentage differences after 10 weeks of Saskatoon berry intake. Bolded values and asterisks (*) indicate *p* < 0.05 for pairwise *t*-tests comparing T0 vs. T10. The calculation method for physical activity index is described in Ref. [11]: unfit (physical activity index = 0) was defined as recreational activity <1–2 times/week and <20 min/session; active (physical activity index = 1) was defined as recreational activity for 1–2 times/week for >20 min/session or >2 times/week and <20 min/session; and fit (physical activity index = 2) was defined as recreational activity >2 times/week and >20 min/session. *p*-values were corrected for multiple testing using the Benjamini–Hochberg method.

**Table 3:** Dietary intake of participants (n = 20) at baseline and after 10 weeks of intervention.

Nutrients/Day	T0	T10	Change (%)	*p*_(FDR)_
Calories (kcal)	1840 ± 689.8	1815 ± 519.1	−1.37	0.871
Proteins (g)	93.10 ± 35.56	82.87 ± 26.22	−10.99	0.162
Carbohydrates (g)	208.3 ± 104.9	234.0 ± 75.53	+12.36	0.294
Total Fiber (g)	17.99 ± 13.06	24.70 ± 10.00	+37.29	0.021 *
Sugar (g)	60.14 ± 55.82	85.17 ± 32.89	+41.61	0.046 *
Total fat (g)	70.18 ± 29.50	64.45 ± 34.92	−8.16	0.422
Saturated fat (g)	23.91 ± 12.02	19.95 ± 13.10	−16.55	0.202
Cholesterol (mg)	436.8 ± 319.0	337.0 ± 371.0	−22.86	0.082
Vit C (mg)	47.73± 58.28	82.22 ± 95.90	+72.26	0.028 *
Vit E (mg)	3.46 ± 2.11	3.07 ± 2.66	−11.28	0.439
Calcium (mg)	557.5 ± 341.4	526.2 ± 252.1	−5.62	0.707
Iron (mg)	12.59 ± 8.25	13.47 ± 8.69	+7.03	0.546
Magnesium (mg)	155.8 ± 87.02	168.4 ± 63.08	+8.09	0.458
Potassium (g)	2057 ± 1001	2377 ± 930.2	+15.54	0.172
Sodium (mg)	2581 ± 998.5	2844 ± 1291	+10.20	0.400
Caffeine (mg)	208.3 ± 481.0	177.1 ± 364.7	−15.00	0.302

Abbreviations: T0: baseline; T10: 10 weeks post-intervention; Vit: Vitamin; Vit A-RAE: Vitamin A—Retinol Activity Equivalent. Bolded values and asterisks (*) indicate *p* < 0.05 for pairwise *t*-tests comparing T0 vs. T10.

**Table 4:** Post-intervention survey and feedback from participants.

Questions	Answers
Did you find it easy to eat one package of the berries every day?	Yes: 81%; no: 19%
What time of the day did you usually eat the berries?	Morning 37%; afternoon 50%; evening 13%
How did you eat the berries? (e.g., in cereal, as a snack, etc.)	Snack: 81%; cereal: 13%; others: 6%
How many days did you miss taking the berries?	0 days: 56%; 1–2 days:38%; 3–4 days 6%
Did you like the texture of the berries?	Liked: 87%; disliked: 13%
Did you like the taste of the berries?	Liked: 94%; disliked: 7%
Did you observe any changes with your health during the study period?	Yes: 60% (weight loss, bowel habit, less sugar craving); no: 40%
Did you notice any changes in bowel habits during your intake of dried berries?	Increased: 62%; no change: 40%
If there were a follow-up study on Saskatoon berries, would you consider participating again or recommend it to your friends or relatives?	Yes: 94%; no: 6%

## Data Availability

The sequencing data were deposited to the Sequence Read Archive (SRA) by National Center for Biotechnology Information under project ID PRJNA1398922. All other data supporting the findings of this study are available upon request.

## Supplementary Materials

The following supporting information can be downloaded at: https://www.mdpi.com/article/10.3390/ijms27083644/s1.
