# Supplementary material for: Effects of Daily Saskatoon Berry Supplementation on Cardiometabolic Health, Gut Microbiota, and Short-Chain Fatty Acids in Healthy Adults
Source: Int J Mol Sci. 2026 Apr 19;27(8):3644. doi: 10.3390/ijms27083644 (PMC13116754; doi:10.3390/ijms27083644)
Supplement: Supplementary file 1 [file ijms-27-03644-s001.zip › ijms-4222050-supplementary.pdf]

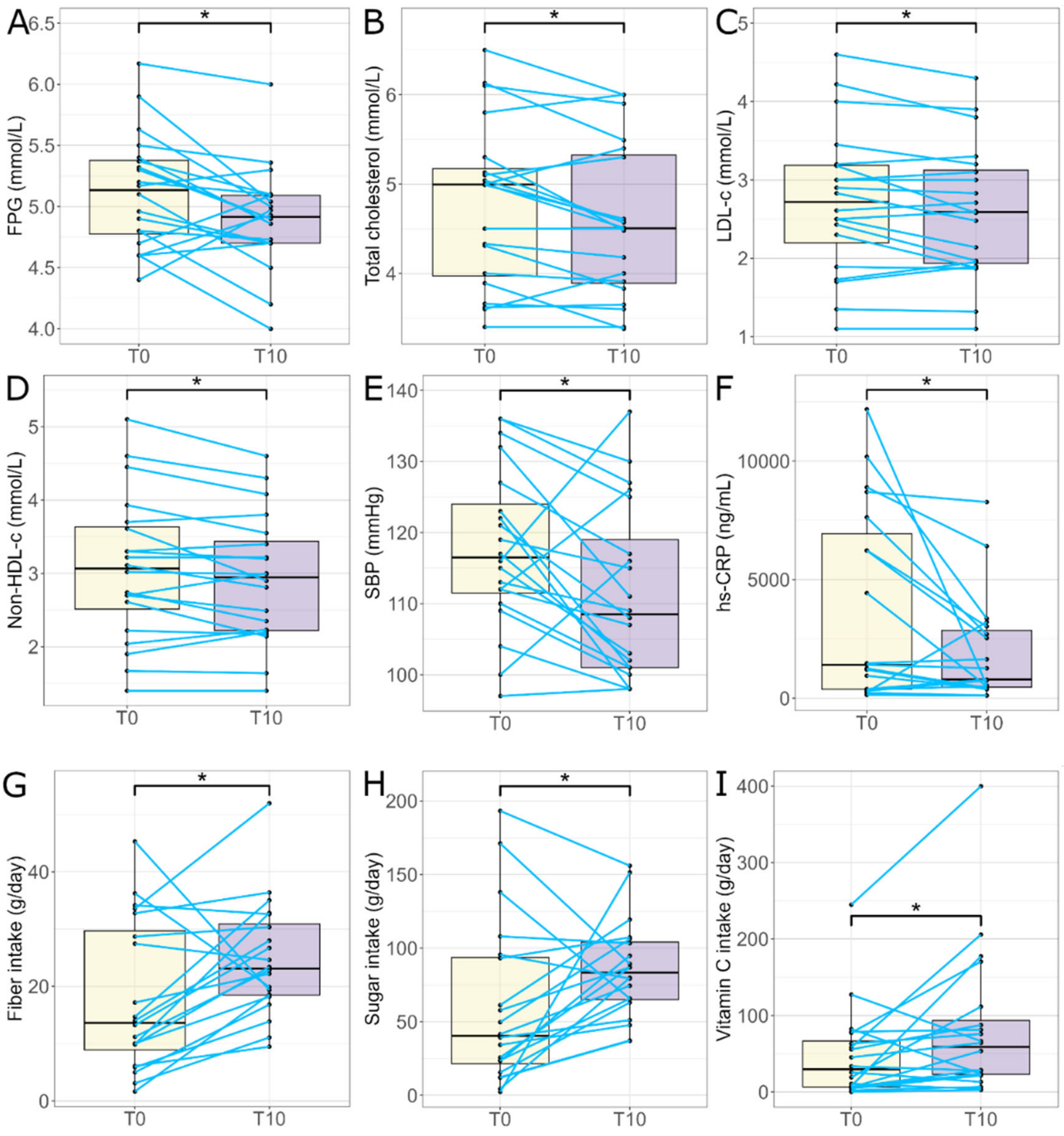

Fig. S1 Boxplots visualizing pairwise comparisons of clinical and dietary variables with statistical significance between pre- (T0) and post- (T10) dietary intervention groups. FPG: fasting plasma glucose; LDL-c: low-density lipoprotein cholesterol; non-HDL-c: non-high-density cholesterol; SBP: systolic blood pressure; hs-CRP: high-sensitivity C-reactive protein. Sugar intake, hs-CRP and vitamin C intake were assessed using the pairwise Wilcoxon signed-rank test, while other variables were analyzed using the pairwise t-test. Blue lines connect data from the same individuals. Pairwise analysis for hs-CRP were conducted using nineteen pairs (n = 38), whereas twenty pairs (n = 40) were included for all other variables. *: $p_{(FDR)} < 0.05$.

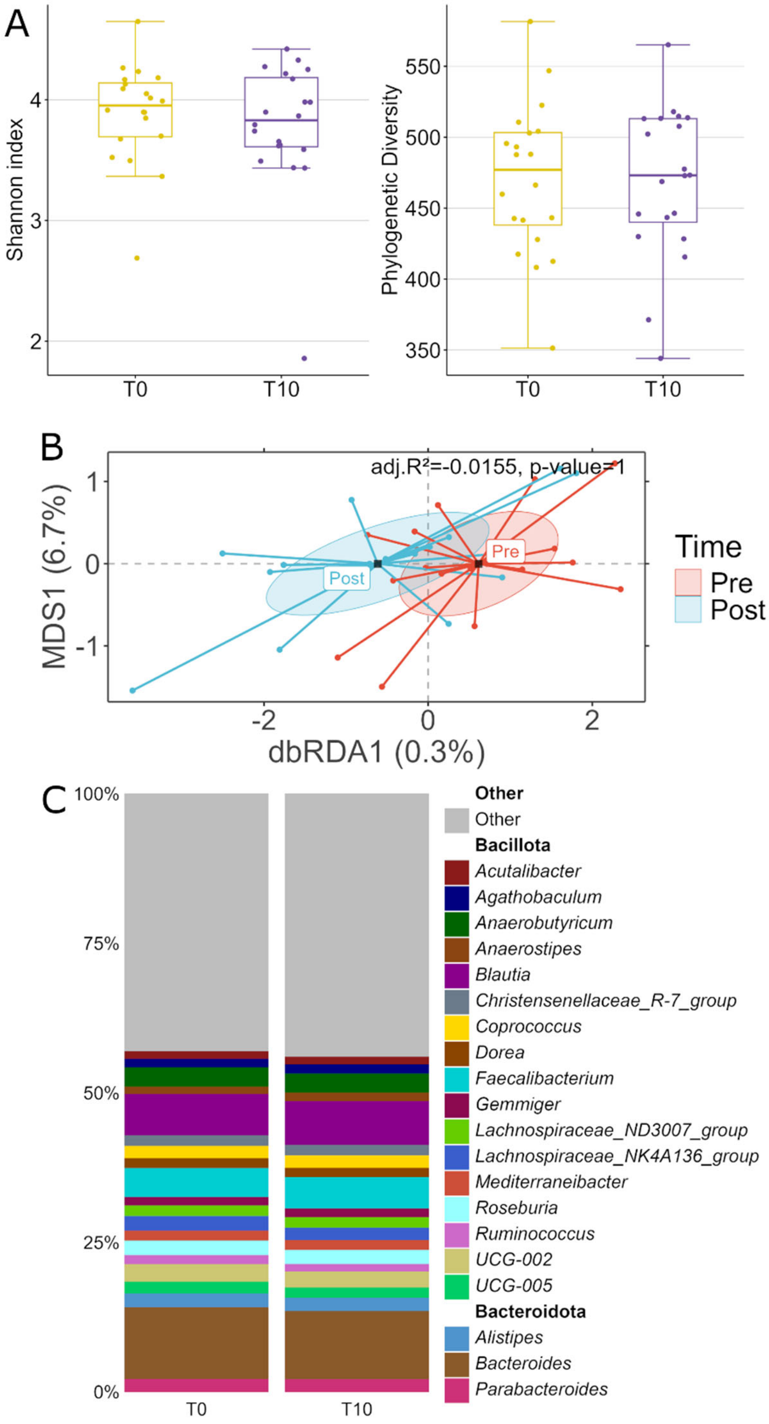

Fig. S2 Gut microbiota diversity and differential abundance analyses at the genus level. (A) α-diversity was assessed using the Shannon index and phylogenetic diversity. Rarefaction was applied based on the lowest sample read counts of 26,568 to compare diversity between pre- (T0) and post- (T10) intervention groups. (B) β-diversity was evaluated using Bray-Curtis dissimilarities and distance-based redundancy analysis (db-RDA). The analysis was adjusted for age and sex covariates with 9,999 permutations used to test significance between T0 and T10. (C) Relative abundance of gut

microbiota at the genus level in the T0 and T10 groups. Data are presented for the 20 most abundant genera; none were significantly different between T0 and T10.
